# Delirium in the intensive care unit: identifying difficulties in applying the Confusion Assessment Method for the Intensive Care Unit (CAM-ICU)

**DOI:** 10.1186/s12912-022-01103-w

**Published:** 2022-11-23

**Authors:** Fernanda Cecília Monroe dos Santos, Adriana Sousa Rêgo, Widlani Sousa Montenegro, Sarah Tarcisia Rabelo Ferreira de Carvalho, Rodrigo Costa Cutrim, Abraão Albino Mendes Júnior, Fábio Henrique Ferreira Pereira, Almir Vieira Dibai-Filho, Daniela Bassi-Dibai

**Affiliations:** 1 Postgraduate Program in Programs Management and Health Services, Universidade Ceuma, Rua Josué Montello, 1, Jardim Renascença, MA CEP 65075-120 São Luís, Brazil; 2 Hospital São Domingos, São Luís, MA Brazil; 3 Department of Physical Therapy, Universidade Ceuma, São Luís, MA Brazil; 4 Postgraduate Program in Dentistry, Universidade Ceuma, São Luís, MA Brazil; 5 Postgraduate Program in Environment, Universidade Ceuma, São Luís, MA Brazil; 6 Postgraduate Program in Physical Education, Universidade Federal do Maranhão, São Luís, MA Brazil

**Keywords:** Nursing, Qualitative study, Intensive care units

## Abstract

**Background:**

Delirium is an underdiagnosed condition and this may be related, among other causes, to the incorrect use of assessment tools due to lack of knowledge about cognitive assessment and lack of training of the care team. The aim of this study was to investigate the difficulties encountered by the nursing team in the application of the Confusion Assessment Method for the Intensive Care Unit (CAM-ICU) in patients on mechanical ventilation.

**Methods:**

This is descriptive study with a qualitative approach in a private tertiary hospital located in northeast Brazil. Data collection took place from July 2018 to January 2019. We included 32 nurses and used face-to-face semi-structured interviews. The recorded data were analysed using content analysis. This study followed the recommendations of the Standards for Reporting Qualitative Research (SRQR).

**Results:**

We identified three major categories: lack of knowledge of professionals, subdivided into deficit in academic formation, difficulty in the differential diagnosis of delirium and delusion, and lack of knowledge about the steps of the CAM-ICU; difficulty in patient cooperation; and lack of adequate training to apply the CAM-ICU.

**Conclusion:**

Nurses have a deficit in academic formation on delirium and need adequate training for the correct and frequent use of the CAM-ICU.

**Supplementary Information:**

The online version contains supplementary material available at 10.1186/s12912-022-01103-w.

## Introduction

Delirium is a common manifestation of brain dysfunction characterized by an acute, transient, and fluctuating course of confusion, with cognitive changes involving memory, perception, and attention. The occurrence of delirium is associated with worse outcomes, such as prolonged hospital stay and mechanical ventilation, increased mortality, and cognitive dysfunction [1, 2].

Risk factors for the development of delirium can be divided into predisposing and precipitating. Among the predisposing factors, the following stand out: age greater than 65 years, male, previous cognitive deficit, depression, visual or hearing deficit, high severity score on admission, alcoholism and smoking, systemic arterial hypertension, malnutrition, and APOE E4 polymorphism. As for the precipitating factors, the following stand out: use of catheters, mechanical containment, sleep deprivation, respiratory disease with hypoxemia, use of benzodiazepines, anemia, hydroelectrolytic changes, use of psychoactive medications, pain, sepsis, and intensive care unit (ICU) admission [3–5].

Delirium is an underdiagnosed condition and this may be related, among other causes, to the incorrect use of assessment tools due to lack of knowledge about cognitive assessment and lack of training of the care team [6]. The specialized literature presents some instruments for the assessment of delirium, with emphasis on Confusion Assessment Method for the Intensive Care Unit (CAM-ICU) [7].

The CAM-ICU is an adaptation of the Confusion Assessment Method, a method described in 1991 by Inouye and colleagues to assess mental confusion in patients outside the intensive care environment. The CAM-ICU was validated in 2001 by Ely et al*.* [8] for patients on mechanical ventilation, presenting a high sensitivity (≥ 93%) and specificity (≥ 98%) for detecting delirium, in addition to presenting high inter-observer reliability (kappa = 0.96).

This is a quick application tool, with an average completion time of 2 min. To apply the CAM-ICU, the patient's level of sedation must be assessed using the Richmond Agitation-Sedation Scale (RASS) [8]. In addition, during the application of the scale, four characteristics should be evaluated: acute changes in mental status or fluctuating course, inattentiveness, disorganized thinking, and altered level of consciousness. The diagnosis of delirium occurs if three of these characteristics are present [8, 9].

However, a study carried out in Poland conducted by Kotfis et al*.* [7] identified worrying results regarding inadequate knowledge about delirium among health professionals working in ICU, with disease monitoring in only 12% of these units. Therefore, considering the clinical context mentioned here, the objective of this study was to investigate the difficulties encountered by the nursing team in the application of the CAM-ICU in ICU patients.

## Methods

### Design and setting

This is descriptive study with a qualitative approach in a private tertiary hospital located in São Luís (Maranhão, northeast Brazil), nationally accredited with the accreditation seal of excellence granted by the National Accreditation Organization and internationally by the Qmentum Accreditation Certification. It is considered a highly complex hospital, with 256 beds, offering services in all areas of intensive care, including major surgeries in vascular surgery, urology, digestive and hepatobiliary surgery, oncological surgery and oncology, clinical and surgical emergencies, cardiology and cardiac surgery, nephrology, neurology and neurosurgery, traumatology and orthopedics.

The present study followed the recommendations of the Standards for Reporting Qualitative Research (SRQR) [10].

### Participants

The first stage of the research consisted of inviting nurses from ICU to schedule the interviews via email and cell phone message. The selection of nurses was based on the database of the intensive care nursing coordination sector, with a list of nurses working in that sector provided. Nurses working in all 5 ICU of the hospital were included.

The inclusion criteria were:

- Nurses of both sexes;
- Working in at least one of the five ICU of the hospital (4 general ICU and 1 cardiac ICU);
- At least one year of experience in the sector.

The exclusion criteria were:

- Nurses who were away from work;
- Nurses who were on vacation during the data collection period;
- Nurses who exercise management or coordination positions without any clinical interaction with the patient.

### Data collection

Data collection took place from July 2018 to January 2019. For data collection, participant observation was used, with notes in a field diary and a semi-structured interview with an initial part for collecting sociodemographic data. The semi-structured interview consisted of open questions, in which the interviewee had the possibility to discuss the proposed topic, without answers or conditions prefixed by the researcher and was carried out in an environment outside the hospital so that there was no embarrassment in the answers. The period for carrying out the interviews was defined by the interviewee, according to their availability of time, lasting 15 min. The interviews were recorded and then transcribed by one of the researchers.

The participant observation comprised the monitoring of nurses during the application of the CAM-ICU scale during the routine, in sporadic shifts. We consider participant observation as the one in which the researcher participates and interferes in the investigated context, with emphasis on the application of the scale by ICU nurses [11].

To carry out non-participant systematic observation, the checklist by Torres et al. [12] was used, containing the observation items that were adopted in this study, such as the difficulty in applying the CAM-ICU scale and insecurity in applying the scale alone. In this systematic observation, the researcher participated in the daily life of nurses in ICU, observing the difficulties experienced and how they behave in front of them, engaging in conversations with one or all of those involved in the situation, in order to understand the group's interpretations.

Participant observation and non-participant systematic observation were carried out in the three shifts and totaled 160 h of recording. Data collection was performed by a hospital nurse.

### Data analysis

As a method of data analysis, we used a qualitative approach through content analysis, as described in previous studies [13, 14], carried out from three stages:

- pre-analysis, which consisted of choosing the documents to be analyzed, resuming hypotheses and the initial objectives of the research, reformulating them in view of the material collected, and in the elaboration of indicators that guided the final interpretation, based on the definition of the nuclei of meaning from the theoretical saturation framework;
- exploration of the material or coding, which was characterized by the transformation of raw data in order to reach the understanding of the core of the text, grouping them into large categories;
- treatment of the results obtained or interpretation, which was based on the proposition of inferences and interpretations foreseen in the theoretical framework.

### Ethical aspects

The present study was approved by the research ethics committee of Universidade Ceuma (opinion number 2,994,607) and followed the Helsinki statements on research ethics.

## Results

This study consisted of 32 nurses. Eight nurses worked in the cardiac ICU and 24 worked in the general ICU. Most of the sample consisted of women aged between 24 and 29 years. Other sample details are described in Table 1. The CAM-ICU has been routinely used for more than 10 years in the hospital of this research.

**Table 1:** Characterization of nurses working in intensive care units (ICU) who were interviewed in the study (*n* = 32)

Variable	Category	n (%)
**Sex**	Female	25 (78.1%)
	Male	7 (21.9%)
**Age**	24–29 years	17 (53.12%)
	30–60 years	15 (46.88%)
**Service time in the ICU**	Up to 2 years	2 (6.25%)
	> 2 to 5 years	17 (53.12%)
	> 5 years	13 (40.62%)
**Highest academic degree**	Master’s degree	1 (3.12%)
	Specialization	31 (96.88%)

Using content analysis, we reached theoretical saturation in the thirty-second interview. Thus, through the analysis of the empirical data collected, it was possible to form three major categories: 1) lack of knowledge of professionals, subdivided into: a) deficit in academic formation, b) difficulty in the differential diagnosis of delirium and delusion, c) lack of knowledge about the steps of the CAM-ICU; 2) difficulty in patient cooperation; and 3) lack of adequate training to apply the CAM-ICU.

When nurses were asked at what point in their academic career (undergraduate or postgraduate) they had heard about delirium, we identified that the vast majority of reports showed that knowledge about delirium occurred in the work environment and, later, in postgraduate courses. In this context, a nurse, when asked about having studied the subject during undergraduate and/or graduate studies, reported:

*“The first time I heard about delirium was inside the ICU. During nursing course at the university, in the intensive care discipline, this subject was never discussed. I was only able to deeply understand what it was about when I did a postgraduate course, in which a professor explained the reason for the occurrence of delirium and how it was treated” (Nurse 13).*

Despite the similarity between the terms in Brazilian Portuguese, there is an etiological difference between delirium (*delirium* in Portuguese) and delusion (*delírio* in Portuguese). When we questioned the nurses interviewed, we evidenced the difficulty of differentiating these two clinical conditions, as it is possible to demonstrate through the speech of the following interviewees:

“The first time I saw a patient presenting confused dialogue I thought he was with delusion and assumed it was due to some medication. It wasn't until my supervisor approached me that I understood that it could be delirium. So she explained to me details of the delirium and why we had to apply the CAM-ICU” (Nurse 17).

“Today I am fully aware of the difference between delirium and delusion, but when I arrived at the service, which coincidentally was at the time of the implementation of the delirium prevention protocol, it was very common for nurses to think that the patient was evolving with a psychiatric condition. Today I know that delirium is a chemical change in the brain, which has nothing to do with delusion” (Nurse 15).

Despite several studies proving that the CAM-ICU is easy to apply and has adequate reliability, we noticed a lack of knowledge about the application steps of this scale. More than half of the interviewees reported difficulty with the RASS, a scale that must be used for the correct application of the CAM-ICU, as reported below:

*“Yes, I have some difficulty applying the CAM-ICU. The first part of the scale is very easy to assess, as I have experience with RASS, although the -3 score on the RASS always leaves me in doubt whether I should proceed or not” (Nurse 14).*

“I always have doubts in the part of the RASS evaluation, when the patient scores -3. The patient is neither awake nor sedated. This part is a little confusing” (Nurse 23).

One of the great difficulties pointed out by the interviewees is the non-cooperation of patients, especially those on mechanical ventilation. Thus, patients are not always collaborative enough to proceed with the steps of the CAM-ICU, as shown in the following report:

*“My biggest difficulty is to apply it to patients on mechanical ventilation, because they are agitated and cannot understand the commands I try to pass on. Even though I know that a possible hyperactive delirium is already being set up there, I often cannot identify using the CAM-ICU” (Nurse 9).*

When asked about the way they learned to apply the CAM-ICU, we noticed two distinct moments in the hospital studied. In the year of implementation of the delirium prevention program (in 2013), there was an extensive training and dissemination campaign on the scale, as shown in the following report:

*“I was hired at the hospital in the year of implementation of the delirium prevention program. We participated in several trainings so that we could actually know how to apply the tool. We were always called attention when we failed to apply the delirium diagnostic scale to patients (Nurse 29).*

As for professionals with less than five years of service, that is, hired by the hospital after the delirium prevention program, we can see changes in the training offered to nurses for the proper use of the CAM-ICU, according to the following reports:

*“The first time I went to apply the CAM-ICU after the initial training was very confusing. I called the supervising nurse to help me, but she couldn't take long because she was also with critically ill patients. So, she ended up applying the scale and I just stood there watching. There wasn't enough training time for me to actually be able to apply the CAM-ICU. I know that the supervising nurse meant no harm, but the dynamics of the service within the ICU does not allow us to learn at the bedside” (Nurse 13).*

*“I think that as the application of the instrument is no longer charged by the coordination of the service, people kind of teach how to apply the CAM-ICU anyway, and when we are going to apply it to patients we are always in doubt, not even the more experienced supervisors really know how to apply the tool. I never felt supported” (Nurse 12).*

## Discussion

Our study observed that the difficulties in using the CAM-ICU were related to a lack of academic formation of nurses to understand delirium, doubts during the application of the CAM-ICU and lack of adequate training of nurses. The CAM-ICU is an important and widely used instrument for the screening and diagnosis of delirium, with adequate inter-examiner reliability values, i.e., the chance of different examiners finding the same result in the application of the instrument is high [8]. However, the CAM-ICU requires adequate training, experience and clinical repertoire from the professional who uses it.

In this sense, a study carried out in an ICU in the Netherlands identified that, after training nurses through several training sessions that included videos to illustrate different states of delirium, there was an increase from 38 to 95% in the frequency of delirium assessment per shift of nursing. In addition, these authors identified that trained nurses are more aware and value delirium as an important clinical problem [6]. In complement, a study carried out in Poland identified that delirium is monitored in only 12% of the ICUs analyzed in the study [7]. In our study, we observed inadequate academic formation of the nurses for delirium and inadequate training in the hospital, which raises doubts and difficulties in the use of the CAM-ICU.

On the role of nurses in patients with delirium, a study conducted by Krupa et al. [15] in Poland identified that nurses have no knowledge of the factors contributing to the development of delirium, are unable to communicate with such patients and, most of all, do not know the consequences of the actions taken. In this way, we emphasize the importance of an adequate diagnosis of delirium and as early as possible by nurses, using the CAM-ICU, RASS or Nursing Delirium Screening Scale (NuDesc) [16], given that a previous systematic review highlights that non-pharmacological nursing interventions may be effective in preventing and reducing the duration of delirium in ICU patients [17]. However, for the proper use of diagnostic instruments (such as the CAM-ICU), a structural training program is necessary [6].

In addition, a study carried out in Denmark identified three main themes in qualitative analysis with nurses and physicians on the use of the CAM-ICU: 1) professional role issues: CAM-ICU screening affected nursing care, clinical judgment and professional integrity; 2) instrument reliability: nurses and physicians expressed concerns about CAM-ICU assessment in non-sedated patients, patients with multi-organ failure or patients influenced by residual sedatives/opioids; and 3) clinical consequence: after CAM-ICU assessment, physicians lacked evidence-based treatment options, and nurses lacked physician acknowledgment and guidelines for disclosing CAM-ICU results to patients [18].

The importance of the proper diagnosis of delirium using the CAM-ICU will serve as a basis for appropriate interventions to be instituted. In this sense, a previous qualitative study identified three main issues regarding the management of delirium: “1) the decision to treat or not to treat ICU delirium based on delirium phenotype; 2) the decision to act based on experience or evidence; and 3) the decision to intervene using nursing care or medications” [19].

Therefore, it is possible to point out that the valorization of delirium as a relevant clinical condition is directly related to the implementation of the use of the CAM-ICU through the systematization of ICU work and adequate training of nurses [6]. In addition, as a suggestion for future studies to elucidate gaps that still exist, we recommend identifying the reliability of the CAM-ICU for trained and untrained nurses, as well as comparing the reports of experienced versus inexperienced nurses about the difficulties in using the CAM-ICU.

As strengths of the present study, we highlight the representative sample from a robust private hospital with accreditations. In addition, the methodology was clear and well defined to ensure reliability (e.g., interviews were recorded for later transcription to avoid loss of information). Regarding transferability, our study was carried out in a highly complex private hospital, with 256 beds and 5 ICUs. However, the data cannot be extrapolated to hospitals with lower complexity (this is a limitation of the study).

Other limitations of this study should be highlighted. We did not analyze nurses’ reports based on ICU type (e.g., general ICU versus cardiac ICU). We did not assess the opinion of other healthcare professionals, as previous studies have done [18, 19].

## Conclusion

Nurses have a deficit in academic formation on delirium and need adequate training for the correct and frequent use of the CAM-ICU.

## Supplementary Information

**Additional file 1.**

## Data Availability

The data and materials in this paper are available from the corresponding author on request.
